# Genomic Stability of Lyophilized Sheep Somatic Cells before and after Nuclear Transfer

**DOI:** 10.1371/journal.pone.0051317

**Published:** 2013-01-08

**Authors:** Domenico Iuso, Marta Czernik, Fiorella Di Egidio, Silvestre Sampino, Federica Zacchini, Michal Bochenek, Zdzislaw Smorag, Jacek A. Modlinski, Grazyna Ptak, Pasqualino Loi

**Affiliations:** 1 Department of Comparative Biomedical Sciences, University of Teramo, Teramo, Italy; 2 National Research Institute of Animal Production, Balice, Poland; 3 Department of Experimental Embryology, Institute of Genetics and Animal Breeding, Polish Academy of Sciences, Jastrzebiec, Poland; The Babraham Institute, United Kingdom

## Abstract

The unprecedented decline of biodiversity worldwide is urging scientists to collect and store biological material from seriously threatened animals, including large mammals. Lyophilization is being explored as a low-cost system for storage in bio-banks of cells that might be used to expand or restore endangered or extinct species through the procedure of Somatic Cell Nuclear Transfer (SCNT). Here we report that the genome is intact in about 60% of lyophylized sheep lymphocytes, whereas DNA damage occurs randomly in the remaining 40%. Remarkably, lyophilized nuclei injected into enucleated oocytes are repaired by a robust DNA repairing activity of the oocytes, and show normal developmental competence. Cloned embryos derived from lyophylized cells exhibited chromosome and cellular composition comparable to those of embryos derived from fresh donor cells. These findings support the feasibility of lyophylization as a storage procedure of mammalian cells to be used for SCNT.

## Introduction

The biological activity of some organisms can be reversibly suspended when intracellular water is either unavailable following exposure to freezing temperatures, or lost as a result of severe water deprivation. For instance, ectothermic animals, such as anurans, and especially the wood frog (Rana sylvatica), can tolerate having their body fluids frozen for two weeks without harm [1], [2] by increasing the synthesis and concentration of glucose in all their organs [3]. Similarly, many small invertebrates can withstand water loss by entering into an ametabolic state, or dormancy, known as anhydrobiosis [4], [5]. Tardigrades (Milnesium tardigradum) and sleeping chironomids (Polypedilum vanderplanki) are the most studied and best examples [6], but the list of desiccation-tolerant organisms encompasses four phyla [7]. The capacity to survive dehydration is conferred by the disaccharide trehalose, a sugar that starts to accumulate in cells once water stress is sensed. Late Embryogenesis Abundant (LEA) proteins, which were originally identified in seeds [8], also play a role in desiccation-tolerance [7]. Sugars and LEA proteins exercise their protective effect against desiccation by binding to lipid membranes and macromolecules to form a glassy weft that reversibly holds their interaction [9].

Physiological insights into the mechanisms underlying freeze tolerance in frogs have provided the scientific basis for the development of robust deep-freezing protocols. Cryopreservation in liquid nitrogen is now routinely used to store cell lines for the most disparate purposes [10], and protocols for freezing mammalian gametes and embryos are essential tools in human and farm animal assisted reproduction programmes [11]. Conversely, dry state preservation using lyophilization is generally favoured for the long term storage of foodstuffs [12], biologically active peptides [13], and microorganisms (e.g. bacteria, viruses and fungi), used for industrial purposes [14]. The dry preservation of living organisms or cells is more challenging, but nonetheless researchers have tried to lyophilize eukaryotic cells. Enucleated cells, primarily erythrocytes [15], were the targets of the first lyophilization trials. Again, the knowledge gained from anhydrobiosis turned out to the crucial factor for success. Trehalose, either added to the freezing medium or endogenously expressed following transfection of the trehalose biosynthetic enzymes in cells, confers a substantial level of desiccation tolerance to mammalian cells [16], [17]. We recently reported that lyophilized somatic cells are reactivated upon injection into enucleated oocytes and generate normal pre-implantation embryos [18]. This finding supports the use of freeze-drying, a technically easy and low-cost strategy for storing cell lines to preserve biodiversity. However, if lyophilized cells are to be used for increasing the effectives of threatened animal population through somatic cell nuclear transfer (SCNT) [19], [20], it has to be proved that a lyophilized mammalian genome retains its full development potential. Therefore, before embarking on an in vivo development trial, we set up a series of experiments to determine the genomic integrity of the dry cells, and particularly to characterize the cloned embryos resulting from them. As expected, lyophilization caused significant DNA damage randomly in the genome. Remarkably, the damage was efficiently repaired by the oocytes and the developmental potential of such embryos, based on chromosome and cell composition, was similar to that of control embryos obtained by SCNT using fresh cells.

## Results

### Freeze-dried lymphocytes are devoid of water

The water activity (a_w_) (at 22°C) of fresh blood lymphocytes suspended in freezing solution was 0.996, while the resulting freeze-dried lymphocytes were devoid of free water, with a_w_ of 0.224.

### Lyophilized cells maintain good nuclear structure, but increased DNA damage

**Figure 1:**
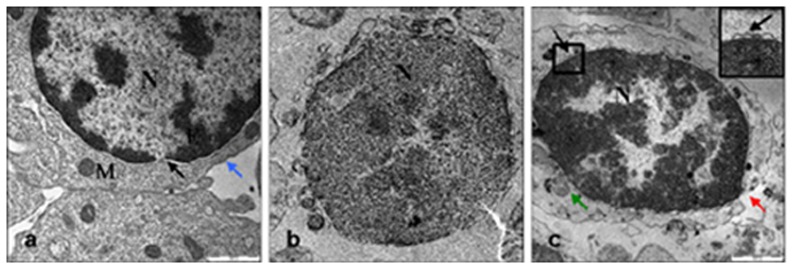
Ultrastructural appearance of fresh (a), frozen (b) and freeze-dried lymphocytes (c). N, nucleus; M, mitochondria, ER, eterochromatin; black arrow, intact nuclear membrane; blue arrow, intact cell membrane; red arrow, broken cell membrane; green arrow, damaged mitochondria Bar = 1000 nm.

Transmission Electron Microscopy (TEM) was used to study the ultrastructure of fresh, frozen-thawed and freeze-dried sheep lymphocytes (Fig. 1). Fresh lymphocytes (Fig. 1A) were characterized by the presence of a large nucleus, dense patches of heterochromatin and intact nuclear membrane, well preserved mitochondria and organelles in the thin rim of cytoplasm surrounding the nucleus, and a few rough endoplasmic reticulum profiles. AS expected, the freeze-dried cells were unviable after rehydration (20% of the live frozen and 0% of the freeze-dried cells). TEM analysis of lyophilized cells revealed that their plasma membrane and mitochondria were degraded (Fig. 1C). As these anomalies were totally absent in fresh samples, and occasionally observed in frozen-thawed cells (Fig. 1B), we hypothesized that they might be due to mechanical injury during desiccation. The nuclear compartment maintained its identity, although significantly shrunken (average diameter: 5.8–6.5 µm fresh to 4.5–5.3 in dry nucleus, data not shown), with a wide chromatin rearrangement with a prevalence of heterochromatin (inset in Fig. 1C).

**Figure 2:**
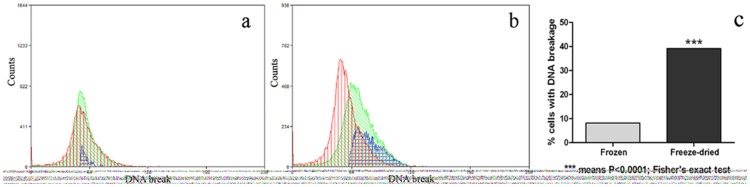
Occurrence of DNA damage in frozen and freeze-dried lymphocytes. (a) DNA breakage in fresh vs frozen lymphocytes and (b) in fresh vs freeze-dried lymphocytes. Left profile: red line = fresh (control) cell counts; green line = frozen lymphocytes cell counts; blue line = proportion of cells with damaged DNA. Right profile: red line = fresh (control) cell counts; green line = freeze-dried lymphocytes, cell counts; blue line; proportion of cells with damaged DNA. (c) Percentage of cells with DNA fragmentation (8.2% frozen and 39.2% freeze-dried cells with DNA damage) (p<0.0001, Fisher's exact test). DNA break of X-axis is the alpha t index.

We then quantified the occurrence of DNA fragmentation in fresh (control), frozen-thawed and freeze-dried lymphocyte samples by flow cytometry after staining them with acridine orange (Fig. 2A, 2B). DNA damage was observed in 8.2% of the frozen and 39.2% of the freeze-dried lymphocytes (p<0.0001) but in none of the controls (fresh lymphocytes) (Fig. 2C).

### Reduced development of embryos reconstructed by SCNT using freeze-dried nuclei

**Figure 3:**
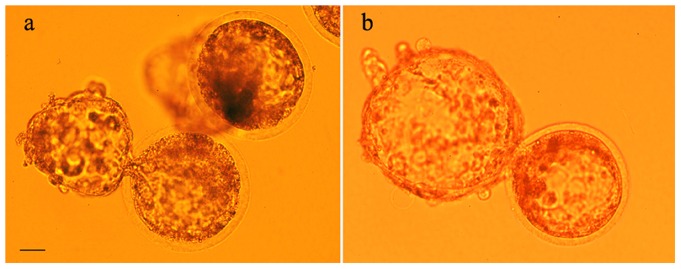
Blastocysts produced by nuclear transfer of fresh and freeze-dried lymphocytes. (a) fresh, (b) freeze-dried lymphocytes as donor cells. Bar = 20 µm.

In order to further explore the functional DNA status of freeze-dried cells, we used them as donor nuclei for SCNT. After being injected into enucleated oocytes, the dry nuclei were reorganized into pronuclei at almost the same frequency as nuclei from fresh lymphocytes (82% vs. 83%), although nuclear swelling was reduced (pronuclear size: 14.99±2.06 µm for fresh and 10.1±0.99 µm for dry nuclei, p<0.02). Conversely, the in vitro development of the reconstructed embryos to the blastocyst stage after SCNT with freeze-dried donor cells was significantly reduced at all stages compared to the embryos obtained by SCNT with fresh donor cells (Table 1) (Fig. 3).

**Table 1:** In vitro development of enucleated oocytes injected with nuclei from freeze-dried and fresh cells.

Donor cells	Cultured	Cleavage	Morula/Cleavage	Blastocyst/Cleavage
Fresh lymphocytes	356	124 (34.8%)^a^	32 (25.8%)^b^	25 (20.2%)^c^
Freeze-dried lymphocytes	825	218 (26.4%)^a^	26 (11.9%)^b^	19 (8.7%)^c^

Embryos were cultured for 7 days. Reconstructed embryos were checked at day 1, 5, 6 and 7 for development;

a p = 0.0035;

b p = 0.001;

c p = 0.0024 (χ^2^ test).

### Normal cellular composition of cloned bastocysts derived from freeze-dried cells

The cell number did not show difference between blastocysts derived from fresh and freeze-dried lymphocytes (**80.11±6 fresh, 78.82±4** freeze-dried) (Table 2). Also the cellular composition (ICM/trophectoderm cell number) was similar in the two groups (Table 2).

**Table 2:** ICM/Trophectoderm differential cell count in blastocysts derived from fresh and freeze-dried lymphocytes.

TOTAL cells	ICM cells	Trophectoderm cells
Fresh NT blastocysts 80.11±6.586	24.00±2.651	56.11±4.452
Freeze-dried NT blastocysts 78.82±4.837	19.73±3.208	59.56±5.620

### DNA damage occurs randomly throughout the genome and is targeted by DNA repair complexes in oocytes reconstructed with freeze-dried nuclei

**Figure 4:**
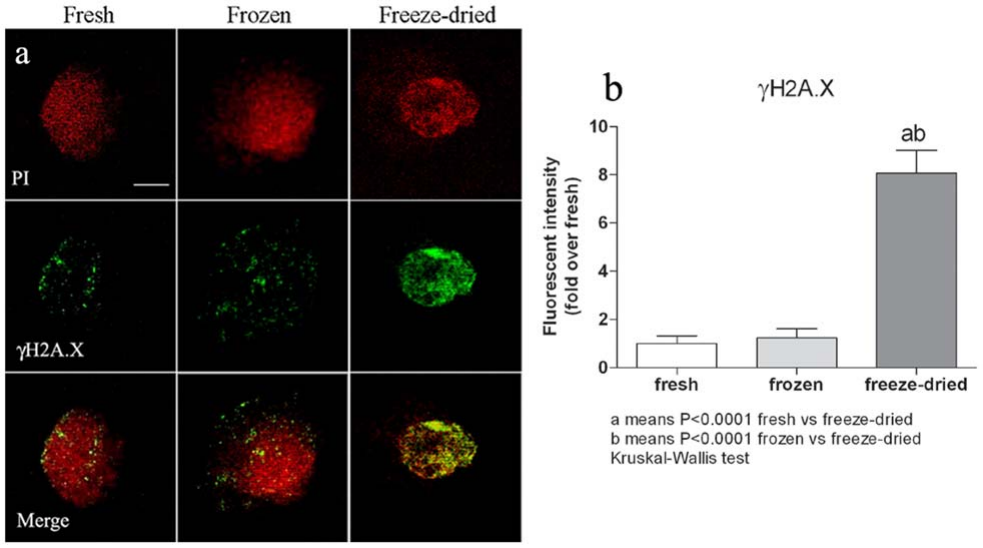
Induction of γH2AX in pronuclei of oocytes injected with nuclei, either from fresh, frozen or freeze-dried lymphocytes. (a) PI = pronuclei stained with propidium iodide; γH2AX = phosphorylated H2AX, a marker of DSBs and, indirectly, of DNA repair; Merge = PI+γH2AX. (b) The γH2AX fluorescent intensity was higher in pronuclei of SCNT-derived embryos using nuclei from freeze-dried cells than in pronuclei of embryos obtained using nuclei from frozen (p<0.0001) or fresh cells (p<0.0001); (Kruskal-Wallis test). Data represent the mean ± SEM of 10 embryos for each group. Scale bar = 10 µm.

**Figure 5:**
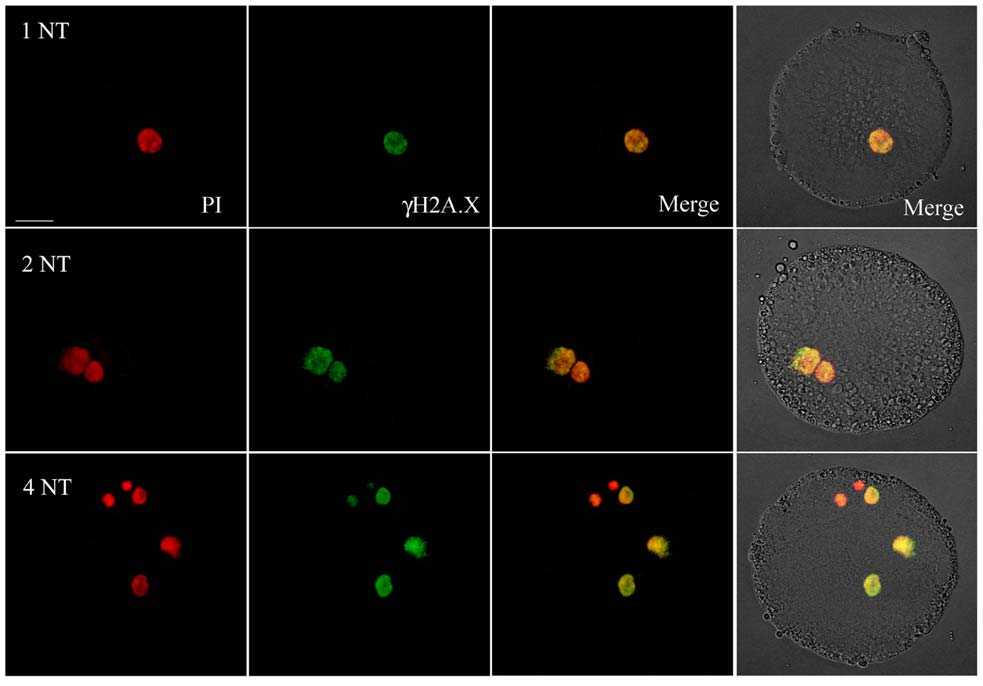
γH2AX immunostaining in pronuclei of oocytes injected with up to four nuclei from freeze-dried lymphocytes. NT = nuclear transfer, 4NT = 4 pronuclei (one fragmentated), PI  = pronuclei stained with propidium iodide; γH2AX = phosphorylated H2AX, a marker of DSBs and, indirectly, of DNA repair; Merge = PI+γH2AX. Scale bar = 20 µm.

In order to determine whether DNA fragmentation had occurred at preferential sites (e.g. inter-nucleosomal loci) as an expression of an apoptotic response induced by DNA lesions, we analysed, by TUNEL assay, pronuclear stage embryos obtained by SCNT using freeze-dried, frozen and fresh lymphocytes (10 embryos for each group) as donor cells. No free 3′-OH ends were detected in any of the samples analysed. This suggests that DNA damage occurs randomly. We then used immunofluorescence to assess the phosphorylation of the histone variant H2AX induced by DNA double strand breaks (DSB). Phosphorylated H2AX (γH2AX) is involved in maintaining the DNA repair machinery and recruiting it to DSB sites [21]. The percentage of γH2AX foci was significantly higher in the pronuclei of oocytes injected with nuclei from freeze-dried cells than from fresh or frozen donor cells (10 SCNT-derived embryos for each group, Fig. 4), while no difference was observed between embryos derived from fresh and frozen nuclei. To empirically quantify the DNA repairing potential of oocytes, we injected up to four nuclei from freeze-dried cells into a subset of oocytes (n = 20) and then assessed the induction of γH2AX. The fluorescence intensity remained unchanged – even in SCNT-derived embryos with four pronuclei (Fig. 5). This suggests that the DNA repair capacity of oocytes is redundant.

### Normal chromosomal composition of early embryos obtained by SCNT using freeze-dried donor cells

**Figure 6:**
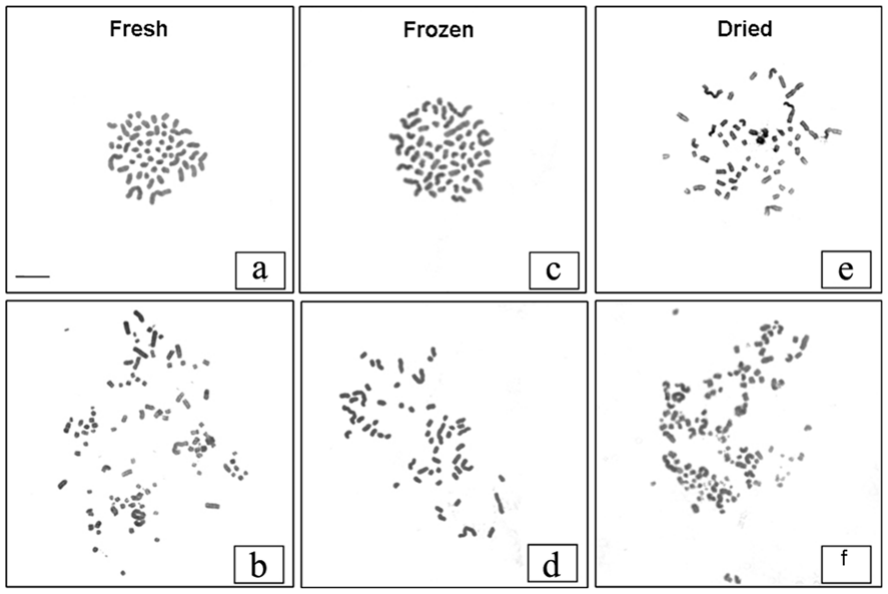
Metaphase spreads of SCNT-derived, 2–4 cell stage embryos. Normal metaphase spreads with 54 chromosomes from embryos obtained by injection of nuclei from (a) fresh, (c) frozen and (e) freeze-dried lymphocytes. Abnormal metaphase spreads from embryos obtained by injection of nuclei from (b) fresh (structural chromosomal abnormalities and chromosome breaks), (d) frozen (55 chromosomes) and (e) freeze-dried lymphocytes (structural chromosomal abnormalities and chromosome breaks).

Embryos obtained by SCNT using fresh and freeze-dried lymphocytes were morphologically equivalent and of good quality (Fig. 3), according to classic embryological standards. To verify the presence of gross chromosomal abnormalities, we prepared metaphase spreads of chromosomes from 2–4 cell-stage SCNT-derived embryos using fresh (n = 10), frozen (n = 12) and freeze-dried (n = 10) donor cells. Only metaphase spreads in which the chromosome number was clearly visible were used (Fig. 6). Most embryos in all groups were euploid, and a similar proportion of abnormal metaphases were detected in the three groups (33% with fresh donor cells; 28.6% with frozen donor cells; and 30% with freeze-dried donor cells). This suggests that the incidence of chromosomal anomalies did not differ in the three groups.

## Discussion

The degree of hydration has a strong influence on the spatial organization of DNA [22]. When hydration is decreased, DNA reversibly shrinks into the A-form [23]. As lyophilized cells have no water, we hypothesized that the freeze-drying procedure would lead to a significant degree of DNA damage and about 40% of freeze-dried cells did in fact have DNA damage. This was mainly due to the drying rather than the freezing procedure, as suggested by the low percentage (about 8%) of DNA fragmentation in the frozen-thawed cells. It is worth noting that the DNA in about 60% of the nuclei of the freeze-dried cells was intact. This was also indicated by the preservation of the nuclear structure and membrane. In contrast to recent work reporting that re-hydrated human haematopoietic cells were viable [24], [25], we found that all the lyophilized lymphocytes were dead after re-hydration. It is likely that some residual water was still present in the cells used in the previous studies [24], [25] as a total absence of water is incompatible with the preservation of the structural integrity of the lipid bilayer [26], as observed in our samples.

In our lyophilization protocol, the disaccharide trehalose was added to the freezing medium, but better protection could probably be obtained by loading cells with the sugar before the freeze-drying procedure, as already tested in human fibroblasts [17]. However, the non-viability of freeze-dried cells does not jeopardize their use as nuclei donor, cell viability is not a constraint for cloning [27].

Having characterized the ultrastructure of the dry cells and the gross DNA damage, we moved on and injected them into enucleated oocytes to further explore their functional DNA status. First, we wanted to see whether DNA fragmentation occurs at preferential sites (e.g. inter-nucleosomal loci) as an expression of an apoptotic drift consequence of DNA lesions. The TUNEL assay indicated an absence of apoptosis in the pronuclei of the injected oocytes. The absence of a 180–200 bp DNA ladder was also noted in the gel shift of the DNA extracted from the lyophilized and control cells (data not shown). DNA fragmentation therefore occurs randomly throughout the genome in dry cells. Given the presence of DNA fragmentation in about 40% of the freeze-dried cells, we then addressed the question of whether the recipient oocytes had the potential to repair it. A strong punctuate of immuno-localization of histone H2AX was found in pronuclear-like structures in oocytes injected with lyophilized cells, while a faint signal was detected in the control and frozen-thawed ones. The quantity and distribution of the H2AX foci found in these latter groups overlap with those described in cloned mouse zygotes [28], where the DNA repairing activity was related to the demethylation of genomic DNA. The abundance of DNA repair mechanisms we have observed, still undiluted even in case 4 nuclei were injected, was unexpected. It is, however, beginning to emerge that high basal H2AX levels are expressed in pluripotent cells, even in the absence of genotoxic stimuli [29]. This finding explains our observation: if a high level of DNA repairing enzymes is a characteristic of pluripotent cells, then the zygote, the totipotent cell *par excellence*, must overexpress the gene, as we have observed.

The prevalent notion is that the oocyte repairing activity is directed toward spermatozoa carrying DNA damaged by physical or chemical stressors [30]–[32]. It may also be possible that DNA breaks are an unavoidable background cost that has to be paid for the thigh packaging of the chromatin in the sperm head, as beginning to emerge [33], and the oocyte has developed a robust strategy to counteract it. Although redundant (as suggested by the high γH2AX expression following the injection of three nuclei), however, the oocyte DNA repair activity failed to repair major damage. This explains the significantly lower number of oocytes injected with nuclei from dry cells that entered the first mitosis. In fact, irradiation of mouse zygotes causes G2-M arrest [34], [35], and a synchronous block at the G2-M checkpoint during the first cell cycle was also observed in sheep oocytes injected with nuclei with major DNA damage [18], [27].

Nuclear reprogramming was also partially compromised in lyophilized nuclei as indicated by the lower frequency of development to the blastocyst stage of SCNT-derived embryos using freeze-dried donor cells. This impaired development is effectively predictable from the morphology of the pronuclei in the first cell cycle. Nuclear swelling results from importing remodelling factors from the oocyte cytoplasm into the transplanted nucleus [36] and is usually related to the extent of genome reprogramming. The pronounced reduction in pronuclear size found in oocytes injected with freeze-dried cells is likely to be due to a limited access to remodelling factors.

The reduced pronuclear size we observed may be a result of the modification of the nucleoplasm and the filaments of the nuclear matrix consequent to the drying process. Lyophilizing F-actin-rich regions results in a cross-linked network of interconnected filaments [37], [38]. Nuclear actin plays an important role in modulating the transcriptional activity of somatic cells transplanted into Xenopus laevis Germinal Vesicles (GV), and hence the extent of genome reprogramming [39]. Nuclear actin may therefore undergo structural changes upon lyophilization/rehydration that hinder the access of nuclear remodelling/reprogramming factors to the chromatin.

1. Finally, the embryos derived from injecting the nuclei of the freeze-dried cells that entered the first mitosis were endowed with a normal chromosome complement and formed very good quality blastocysts, with a cell composition similar to control blastocysts. These embryos probably have a developmental potential that is comparable in every way to that of control cloned embryos. The proportion of cloned embryos developing to the blastocyst stage is significantly lower than in our previous report [18]. The sources of the cells are most likely responsible for these differences: granulosa cells were used in the first report, whereas lyophilized lymphocytes, less efficient as nuclei donors for cloning [40] were used in the present work. The number of blastocysts obtained from lyophilized cells is, however, higher than in a previous report on the mouse [41]. Species specific differences in the cloning efficiency and/or a less efficient lyophilization of the mouse cells - no data are available on the state of DNA after rehydration - might be responsible for these differences.

We have quantified and partially characterized the extent of genomic damage caused by freeze-drying, and found that a good part of it can be corrected by oocyte DNA repair activity. Although lyophilization partially reduces nuclear reprogramming, the embryos that manage to go through the first cell cycle develop to the blastocyst stage, and have a normal chromosome and cellular composition. The ultimate test for these embryos remains the capacity to develop into viable offspring upon transfer into suitable foster mothers.

## Methods

All chemicals were purchased from Sigma, unless otherwise stated.

### Cell collection and freeze-drying

Peripheral blood lymphocytes were isolated from Sarda breed ewes through Ficoll-Paque density gradients and then freeze-dried. Sampling was performed by a veterinarian previously authorized by the inter-athenaeum ethics committee Comitato Etico Interateneo per la Sperimentazione Animale – CEISA - (http://www.unich.it/unichieti/appmanager/federati/CEISA). Our Ethics Committee CEISA specifically approved these experiments. Briefly, 10 µl aliquots of blood lymphocytes (10^6^ cells/ml) were suspended in a freezing solution (Ca^2+^/Mg^2+^-free PBS supplemented with 0.1 M trehalose and 12.5% BSA) and dropped onto a pre-cooled (−196°C) metal plate immersed in liquid nitrogen. The drops of frozen samples were transferred in small pre-cooled vials, placed on a pre-cooled freeze-drier (Freezone Plus 6, Labconco, USA) and lyophilized for 24 hours. The vials were then vacuum-sealed and stored at room temperature (RT) (23–25°C) in the dark until used.

### Measurement of water activity

Water Activity (a_w_) is a measurement of water energy, which indicates the amount of free water in a sample. a_w_ is equivalent to Equilibrium Relative Humidity (ERH), which is the ratio of the water vapour pressure of a sample to the water vapour pressure of pure water at the same temperature. Samples with no free water will have an a_w_ of 0.000, while a sample of pure water will have an a_w_ of 1.000. A_w_ in peripheral blood lymphocytes before and after freeze-drying was calculated with the chilled-mirror dew point technique using an AquaLab CX-2 water activity meter (Decagon Devices Inc.) according to the manufacturer's instructions.

### Rehydration

Immediately before nuclear transplantation, 1 ml of Ca^2+^/Mg^2+^-free PBS was added to each vial of freeze-dried cells. After rehydration, the cells were washed with PBS and suspended in 7% PVP (polyvinylpyrrolidone) (w/v) in 280 mOsm KCl solution for nuclear transfer. The viability of the rehydrated cells was evaluated with Live/Dead Cell Double Staining Kit (Calcein-AM/propidium iodide) staining.

### Transmission Electron Microscopy (TEM)

The morphology of fresh, frozen and freeze-dried lymphocytes was assessed by TEM. The cells were washed twice with PBS and fixed in glutaraldehyde (2.5% in 0.1 M cacodylate buffer, pH 7.4) for 24 h. After washing in ddH2O, the cells were post-fixed in 2% OsO4 in ddH2O for 4 h and washed three times in ddH2O. The cells were then dehydrated through a graded series of ethanol solutions (30%–10 min, 50%–15 min, 70%–24 h, 80%–10 min, 96%–10 min, 100%–10 min, and acetone – twice for 15 min) and were infiltrated with graded concentrations of EPON resin in 100% acetone (1:3–20 min, 1:1–24 h, 3:1–2 h), infused twice for 1 h in pure EPON resin and polymerized at 65°C for 24 h. Next, 60 nm sections were prepared and examined using a LEO 912AB electron microscope. The images were captured using a Slow Scan CCD camera (Proscane) and EsiVision Pro 3.2 software (Soft Imaging Systems GmbH).

### Analysis of DNA damage by flow cytometry

The assessment of the chromatin structure was performed using flow cytometry [42]. After partial DNA denaturation (pH = 1.5), the samples were stained with the metachromatic fluorochrome acridine orange (Ex/Em = 488/525 and 615 nm). Only fragmented strands were opened into single stranded DNA after partial denaturation. The intensity of the green (515–530 nm) (intact double stranded DNA) and red (>630 nm) (fragmented, single stranded DNA) fluorescence was measured using a flow cytometer (DAKO Galaxy, DAKO, Denmark). Approximately 15000–25000 cells were acquired for each cell group at a flow rate of 400–500 events/s (in histogram alpha t index is the value DNA break of X-axis). The alpha t index (alpha t = red/green+red fluorescence), which quantifies the extent of DNA breaks, was calculated for each analysed cell and shown on a histogram. Cells with abnormal chromatin structure showed a distinct shift of the alpha t value.

### Oocyte maturation

Oocytes were matured in vitro in bicarbonate-buffered TCM-199 medium (Gibco) (275 mOsm) containing 2 mM glutamine, 100 mM cysteamine, 0.3 mM sodium pyruvate, 10% foetal bovine serum (FBS) (Gibco), 5 mg/ml FSH (Ovagen), 5 mg/ml LH, and 1 mg/ml estradiol in a humidified atmosphere of 5% CO_2_/air at 39°C for 24 h (Ptak G. et al, 2002).

### Oocyte enucleation and nuclear transfer

The oocytes were incubated in Hepes-buffered TCM-199 medium containing 4 mg/ml BSA, 7.5 mg/ml Cytochalasin B and 5 mg/ml Hoechst 33342 in an incubator for 15 minutes. Enucleation was carried out in Hepes-buffered TCM-199 medium with 0.4% (w/v) BSA and Cytochalasin B by using a Narishighe micromanipulator. The enucleated oocytes were left to recover from the Cytochalasin B treatment and then directly injected with nuclei from fresh, frozen or freeze-dried lymphocytes. The reconstructed oocytes were activated in Hepes-buffered TCM-199 medium containing 5 mg/ml Ionomycin for 5 minutes and then incubated in SOF medium plus antibiotics and 0.8% BSA containing 10 mM Dimethylaminopurine and 7.5 mg/ml Cytochalasin B for 3–5 hours.

### Embryo culture

The reconstructed embryos were transferred into 20 µl drops of SOF enriched with 1% (v∶v) basal medium Eagle (BME)-essential amino acids, 1% (v∶v) minimum essential medium (MEM)- nonessential amino acids (Gibco), 1 mM glutamine, and 8 mg/ml fatty acid-free BSA (SOFaaBSA). The embryos were maintained in a humidified atmosphere of 5% CO_2_, 7% O_2_, 88% N_2_ at 39°C, and the medium renewed on day 3 and day 5 of culture (Ptak G et al, 2002).

### Differential staining of blastocysts

Blastocysts obtained from NT of fresh and freeze-dried lymphocytes were incubated in 500 ml of solution 1 (PBS with 1% Triton X-100 and 100 mg/ml propidium iodide) for 20 s. Subsequently they were transferred to 500 ml of solution 2 (100% ethanol with 25 mg/ml bisbenzimide Hoechst 33258) and stored at 4°C overnight. Blastocysts were then mounted onto a microscope slide and cell counting was performed directly on an inverted fluorescence microscope.

### TUNEL assay

The ApopTag Fluorescein In Situ Apoptosis Detection Kit (Chemicon International) was used according to the manufacturer's instructions. At 10–12 h after oocyte activation, the pronuclear-stage embryos were fixed in 1% paraformaldehyde (PFA) for 15 min, washed 3 times in PBS+0.4% PVP and then permeabilized with 1∶2 (v∶v) ethanol∶acetic acid solution at −20°C for 20 min. After two washes, equilibration buffer was added at RT for 10 s. The embryos were then incubated in the TdT enzyme solution in a humid chamber at 38.5°C for 1 h and the reaction was blocked with Stop/Wash buffer. After 3 washes, the pronuclear-stage embryos were incubated in the staining solution in the dark at RT for 30 min, counterstained with 5 µg/ml propidium iodide (PI), mounted on glass slides and examined with a confocal fluorescence microscope (Radiance 2000, Biorad).

### γH2AX immunostaining

The essay on DNA repairing activity of the oocyte was concentrated on the zygotic stage, where the DNA repairing activity reaches its maximum [28].

At 10–12 h after oocyte activation, the zona pellucida of the pronuclear-stage embryos was removed by incubation in 0.5% (w/v) pronase (Calbiochem 537088) and acid Tyrode's solution for 30 s. The embryos were then fixed in 4% PFA for 30 min, permeabilized with 0.1% (v/v) Triton X-100 in PBS for 30 min and transferred in blocking solution (3% BSA/0.05% Tween 20 (v/v) in PBS) for 1 h (all at RT). The embryos were then incubated with the primary rabbit anti-γH2AX antibody (1∶500) (Millipore) at 4°C overnight. After 3 washes in blocking solution, the secondary FITC-conjugated goat anti-rabbit IgG (1∶500) was added for 30 min at RT. The nuclei were counterstained with 5 µg/mL PI. Images were captured using a confocal fluorescence microscope (Radiance 2000, Biorad). The fluorescence intensity was measured using Image Measurement and Analysis Lab software (IMAL 3.5.10.d). The γH2AX signal intensity of every pronucleus was normalized to the DNA signal (PI) before proceeding to compare the groups (fresh, frozen and freeze-dried NT).

### Chromosome analysis in 2–4 cell stage embryos

At 30–35 h after oocyte activation, the 2–4 cell stage embryos were transferred in SOFaaBSA medium containing 0.05 µg/ml colcemid in order to induce metaphase arrest, and cultured for another 7–8 h. The embryos were then transferred into hypotonic citrate solution (1∶1 30% FBS: 1% sodium citrate) at RT for 3 min and fixed in a 3∶2∶1 solution of H2O∶methanol∶acetic acid at −20°C overnight. The metaphases were spread on slides by using a cooled 1∶1 solution of methanol∶acetic acid and stained with 4% (v∶v) Giemsa. A karyotype was considered normal if it was composed of 54 chromosomes with no structural aberrations.

### Statistical analyses

The Fisher's exact and χ^2^ tests were used to compare quantitative data on DNA damage, in vitro development and frequencies of chromosomal anomalies. Fluorescence intensities were analysed using the Kruskal-Wallis test. The data reported in this paper are the mean (±SEM) for each group. Probability values less than 0.05 were considered to be statistically significant. Statistical analyses were performed using GraphPad Prism 5.0 software.
